# Machine Learning Driven Mental Stress Detection on Reddit Posts Using Natural Language Processing

**DOI:** 10.1007/s44230-023-00020-8

**Published:** 2023-03-30

**Authors:** Shaunak Inamdar, Rishikesh Chapekar, Shilpa Gite, Biswajeet Pradhan

**Affiliations:** 1 AIML Department, Symbiosis Institute of Technology, Symbiosis International (Deemed University), Pune, India; 2 Symbiosis Center for Applied AI (SCAAI), Symbiosis International (Deemed University), Pune, India; 3 Centre for Advanced Modelling and Geospatial Information Systems (CAMGIS), School of Civil and Environmental Engineering, University of Technology Sydney, Sydney, NSW 2007 Australia

**Keywords:** Stress analysis, ELMo embeddings, Machine learning, Natural language processing, BERT, TF-IDF

## Abstract

People’s mental conditions are often reflected in their social media activity due to the internet's anonymity. Psychiatric issues are often detected through such activities and can be addressed in their early stages, potentially preventing the consequences of unattended mental disorders like depression and anxiety. In this paper, the authors have implemented machine learning models and used various embedding techniques to classify posts from the famous social media blog site Reddit as stressful and non-stressful. The dataset used contains user posts that can be analyzed to detect patterns in the social media activity of those diagnosed with mental disorders. This paper uses different NLP (Natural Language Processing) tools such as ELMo (Embeddings from Language Models) word embeddings, BERT (Bidirectional Encoder Representations from Transformers) tokenizers, and BoW (Bag of Words) approach to create word/sentence data that can be fed to machine learning models. The results of each method have been discussed. The results achieved a top F1 score of 0.76, a Precision score of 0.71, and a Recall of 0.74 using only the preprocessed texts and machine learning algorithms to classify the posts. The results achieved by this paper are significant and have the potential to be applied in real-world scenarios to analyze mental stress among social media users. Although this paper focuses on data from Reddit, the techniques used can be transferred to similar social media platforms and could help solve the growing mental health crisis.

## Introduction

The field of stress analysis and sentiment analysis of posts on microblogging sites has been blossoming in recent years. Mental stress leads to many health problems, and it is crucial to identify such cases and provide support. It is observed that high exposure to mental stress results in negative behavioral changes, both mental and physical [1]. In the past, stress analysis has been explored on data from microblogging sites like Twitter and Facebook. However, anonymized sites with longer-form content help give users more freedom in expressing their thoughts and underlying stress. This research provides insight into the topics and characteristics that stress people from all over the world by conducting stress analysis on Reddit posts and identifying indications of stress. This could be used as an opportunity to transform the mental health conversation and help early intervention for depression. There is a clear need for systems for early detection of stress and mental disorders.

This paper considers posts from various subreddits on the popular social media platform Reddit. The dataset [2] contains about 2800 unique texts for training. While this is a good number to make a somewhat accurate analysis, comparing it with the actual social media traffic daily and yearly shows that it is negligible. Around 470,000 comments are made daily on Reddit alone. People make these comments with highly varying backgrounds and ideologies. Apart from Reddit, statistics from the popular microblogging platform, Twitter, show that nearly 500 million tweets are made daily and about 200 billion tweets per year. Social media sites provide an emotional outlet for many people to vent their feelings in a virtual world. The abundance of text posts helps identify which factors signify stress and whether a user is displaying signs of mental stress. In this paper, we have performed experiments to help identify stressful posts on social media using machine learning algorithms. The main contribution of this paper is the significant results achieved by using various embedding techniques in combination with popular machine learning models for text classification and identification of stress.

The main highlights of this paper are:

This paper uses the Dreaddit dataset [2] to identify mental stress in social media blog posts by analyzing self-reported cases and uses different embedding techniques and Machine Learning algorithms to train a model that can identify stressful and non-stressful posts on the social media platform Reddit.

The paper compares different natural language processing (NLP) and embedding techniques like BERT, TF-IDF and Word2Vec and popular machine learning models for the classification of stressful posts on Reddit. Finally, a model is trained using the labeled corpus to identify stressful and non-stressful texts and accurately predict when a particular post indicates mental stress.

The paper is organized as below. The paper first explores the previous works in stress analysis and discusses the results obtained previously by the authors of the chosen dataset in Section 2. Section 3 presents the proposed method of experimentation on the Dreaddit dataset and the details about the dataset. This section also describes the models used and the approach to this problem. Sections 4 and 5 present and discuss the results achieved by the authors. In Section 6, the authors present the conclusion, future scope, and limitations.

## Literature Review

### Stress Analysis on Social Media

This section presents a detailed literature review on stress analysis using language processing techniques on social media. Over the last decade, mental health diagnosis over social media has grown into a large field of study. In the past, there have been studies analyzing mental disorders by analyzing word usage in various fields, for instance, poetry [3], college essays [4], and the narrative style of participants [5]. In a paper published by Tsinghua University in China, several behavioral factors have been considered while creating a dataset from an existing pool of Twitter posts [6], thus focusing on developing a hybrid detection model. Social media has become an outlet for people to express themselves in the recent past. Studies using data from social media platforms and identifying toxic speech using Deep Learning methods and even self-reported signs of depression from users using neural networks [7] are making breakthroughs in NLP. These works classify users’ mental health conditions who post in specific categories or subreddits. In another work, Pirina et al. (2018) dealt with identifying depression based on user activity and interaction [8]. Studies have focused on the chronic stress-related diseases caused by the urban environment [9], showing that many patients suffering from such diseases have expressed the same either directly or indirectly on social media platforms. The mental state developed in adolescents, known as Fear Of Missing Out [10], has caused several emotional symptoms, leading to chronic or acute stress diseases. The same study has also shown that this FOMO (Fear of missing out) is directly related to the sensitivity of the users to social media addictions. This further increases their need to consume more content from social media, searching for inspiration or anything to boost their self-confidence [11]. Instead, they lose self-esteem, leading to more degradation of mental health.

Some restrictions require the datasets to be anonymized before they can be accessed. While this is a burdensome process, using data from a social media site like Reddit, where users are anonymous, makes it a convenient data source. In Guntuku et al. [12], the authors have considered various factors that should be considered while analyzing social media posts to detect patterns indicating stress or other mental conditions. However, their dataset is limited to 600 people, and all of them belong to the USA. The general trends among people of different countries will vary significantly and will be governed by that country’s culture. As a result, what applies to the people of one country in terms of psychological behavior may not be accurate for similar people from a different country who have adapted to a different culture. Lin et al. [13] proposed a deep learning model to detect users’ psychological stress in a separate work. They mainly consider the content attributes such as text and images from the post and the user scope or reach of the post. The authors tackle the problem of combining the various attributes by using autoencoders. Their method for extracting features from posts is novel, but the authors have not used a labeled dataset and labeled their data using linguistic attributes that often overlook hidden traits.

Ansari et al. (2021) [14] used the Dreaddit dataset to tackle the limitation of insufficient, labeled data by using augmentation techniques on classification. The paper uses EDA (Easy Data Augmentation), AugBERT, and Back Translation methods for data augmentation and used Random Forest (RF), support vector machines (SVM), and logistic regression (LR) classifiers to test their methods. The highest results obtained by this paper on the original dataset were an F1 score of 0.75, a precision score of 0.68, and a Recall of 0.84. These scores have been achieved using a combination of RF classifier, Word2vec embeddings, and term frequency and inverse document frequency (TF-IDF) vectors. In our paper, TFIDF vectors have been used along with a bag of words (BoW) embeddings. Turcan and McKeown [15] used the Dreaddit dataset with an explainable framework for stress detection, which can provide help in the form of a therapy chatbot or other deployable models for providing psychological help. In this paper [15], the authors worked on several datasets using Bidirectional Encoder Representations from Transformers (BERT) models to label emotions on posts. Their work obtained the highest accuracy of 82.49 using the MultiAlt model on the Dreaddit dataset. Muñoz et al. (2022) showed a comparative analysis of embedding techniques using LR, SVM and Stochastic Gradient Descent (SGD) models and achieved Fscores of over 80% [16]. Yang et al. (2022) achieved an F1 score of 83.5% on the Dreaddit dataset [17] using a KC-Net framework.

In post –Covid-19 times, Saura et al. (2022) [18] analyzed the Twitter posts to understand the challenges of remote work and new opportunities using Natural Language Processing. The authors used TextBlob combined with RF, SVM, LR, and Naïve Bayes (NB) classifiers to perform sentiment analysis on the Twitter data collected from the Twitter API. The highest result obtained by the authors was the accuracy of Linear SVC, which gave an accuracy of 0.87, followed by LR which gave an accuracy of 0.83. The authors also explored the topic modeling using LDA to identify remote work's main opportunities and challenges. This was done by analyzing frequent word usage. Low et al. (2020)[19] explored the trends in language features of Reddit posts during the pandemic and identified whether or not a specific post was posted on a particular subreddit. Their paper uncovered that some subreddit posts had started to become more similar during the pandemic due to anxiety amongst the public due to the uncertainty in the COVID-19 era. The authors focused more on individual post data to classify the posts. This was achieved using NLP techniques and with the help of machine learning (ML). Further, the authors used different embedding techniques combined with vectors and ML models to achieve significant results in classifying posts and identifying the factors affecting stress.

### Word Embedding Techniques

#### ELMo

To convert tokens to features, the authors made use of ELMo. Embeddings from Language Models (ELMo) are representations of embeddings that are based on a bidirectional language model [20].The formation of contextual word embeddings using ELMo enhances sample efficiency and gives a higher F1 score using a lower number of epochs.

#### BERT

BERT was introduced in 2018 by researchers at Google [21] and is primarily used for question answering and sentence prediction. In this study, we used BERT to tokenize the texts of social media posts and convert them into features to train machine learning classifiers. The BERT Large tokenizer was employed to create a feature list of inputs for training, which is particularly useful as it creates contextual word embeddings that aid in analyzing whether or not the words indicate stress within the context of the sentence [22]. BERT, along with fastText embeddings, has also been applied to detect toxic speech on social media platforms [23].

#### Bag of Words Word Embeddings

A bag of words model extracts features from a text, generates a set of vocabulary or known words, and quantifies the extracted vital point from the target data to one of the visual words [24]. The generation of a BoW model involves simply creating a list of all words present in a given data. Further processing can then be applied to it, such as stop words removal, lemmatization, stemming, etc., to prepare the vocabulary suitable for accurate modeling [25].

#### TF—IDF Vectors

This technique is based on a statistical analysis that evaluates the relevance of a word or a term to a document by analyzing a set of documents [26]. It is an accurate measure for determining how relevant the given the word is to the machine learning model to be trained [27]. The authors have used the TF-IDF vectorizer from Python’s scikit-learn library [28].

### Classification Models

In a recent work, [29] explored a few popular machine learning algorithms such as DT and RF which work on a tree traversal method to predict or classify a test data point. In simple words, their work like an if-else condition statement. This means these models will only be able to classify a data point to a label if every feature already exists in the training dataset.

In another work, [30] stated that the problem arises with the k-nearest neighbor (KNN) algorithm because the data is text-based. There are far too many ways of describing a particular situation in the English language. The perspective problem cannot be eliminated in the preprocessing stages. Because of this, plotting data points on an n-dimensional graph to go ahead with KNN becomes pointless.

Another problem with the KNN algorithm is its instance-based nature [31], which makes it undesirable for use in larger datasets. Specific techniques can reduce the vector size and increase accuracy [32].

#### Support Vector Machines

In this paper, we used the Linear SVM model for the classification of social media posts into stress and non-stress categories. The input to SVM was the feature matrix extracted by the embeddings and annotated labels indicating the effect of mental stress. SVMs are known to be effective in classification tasks with smaller datasets and have been proven to be effective in this study as well [33], [34]. The algorithm splits the input examples to create a hyperplane that maximizes margin and minimizes empirical risk.

#### XGBoost

Extreme Gradient boosting (XGBoost) algorithms have been widely used for tasks like image classification [35] and diagnosing diseases such as Kidney disease [36] and heart disease [37]. This boosting algorithm was introduced in 2015 [38] and was implemented as a gradient boosting algorithm.

#### Logistic Regression

LR is a standard algorithm used in various machine learning applications which focuses on binary classification using the sigmoid value function to show how close a target data point is to one of the two possible classification labels. This was chosen because of its popular usage in various classification methods [39], [40] and high accuracy.

#### Research Gaps

The current research on the classification of text data for detecting mental stress in social media posts has not explored the full potential of embedding techniques like ELMo vectors and BERT, in combination with popular machine learning models like LR, SVM, and XGBoost. This paper addresses this research gap by presenting a novel approach that combines ELMo vectors with ensemble and supervised learning algorithms to accurately detect mental stress in Reddit posts.

Moreover, while previous research has focused on other social media platforms, our study focuses on Reddit, which has comparatively lower exposure to stress analysis, and hence represents an important research gap. Our approach has achieved significant results, outperforming existing state-of-the-art methods.

However, our study does not consider the cultural differences between the authors of the posts, which could impact the classification results. Despite this limitation, our research demonstrates the potential of our approach to improve the current mental health support infrastructure on social media platforms and improve the lives of many users.

Overall, this study contributes significantly to the existing literature by highlighting the potential of embedding techniques like ELMo vectors and the importance of considering the platform-specific characteristics when developing stress detection models. Our work represents an important step towards improving mental health support on social media platforms, and future research should build upon our findings to explore these research gaps further.

## Data and Methodology

This section describes the dataset used, the detailed methodology of the proposed research idea with an architectural diagram, and the implemented models.

### Data Used

The Dreaddit dataset is commonly used for stress analysis, social media activity analysis, or generic NLP exercises [41]. The dataset contains valuable metadata and the actual text content, which proves to be particularly valuable for our purpose. Specific points such as tone and anger are beneficial. Sometimes, mere text operations like keyword extraction and further processing to classify a positive or negative mood are not enough. The results can vary heavily if the author of the text in question has applied abstract concepts of the English language, like passive aggression or subtle humor.

Our dataset consists of a text corpus containing the Reddit post's body, the name of the community or subreddit, a label field, and various other fields such as the post karma and timestamp. Many of these parameters are not relevant to our main aim of classifying Stressful and Non-Stressful posts. These parameters are dropped. The following preprocessing step includes cleaning up the text using various NLP methods. The text is first rid of all the stop words and punctuations. It is then tokenized using Rake. These tokens are further formed into features using ELMo embeddings.

Since the input dataset has less than 10000 lines, therefore, traditional machine learning models were used than the deep learning techniques as they work better on larger datasets [42].

**Fig. 1:**
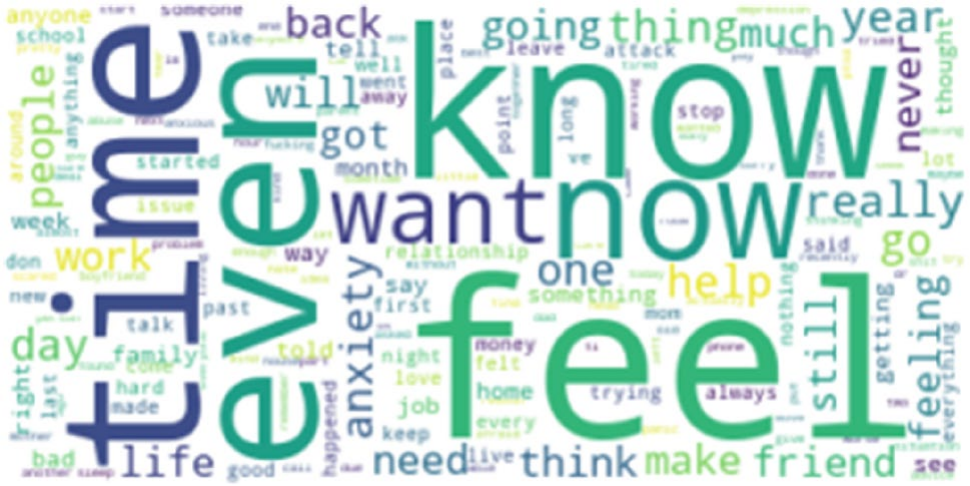
Word cloud depicting stressful posts

**Fig. 2:**
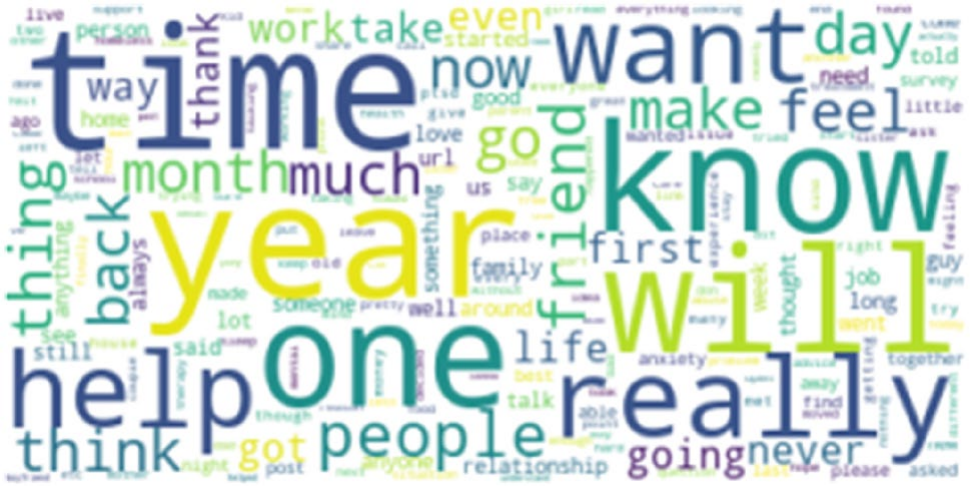
Word cloud depicting non-stressful posts

**Fig. 3:**
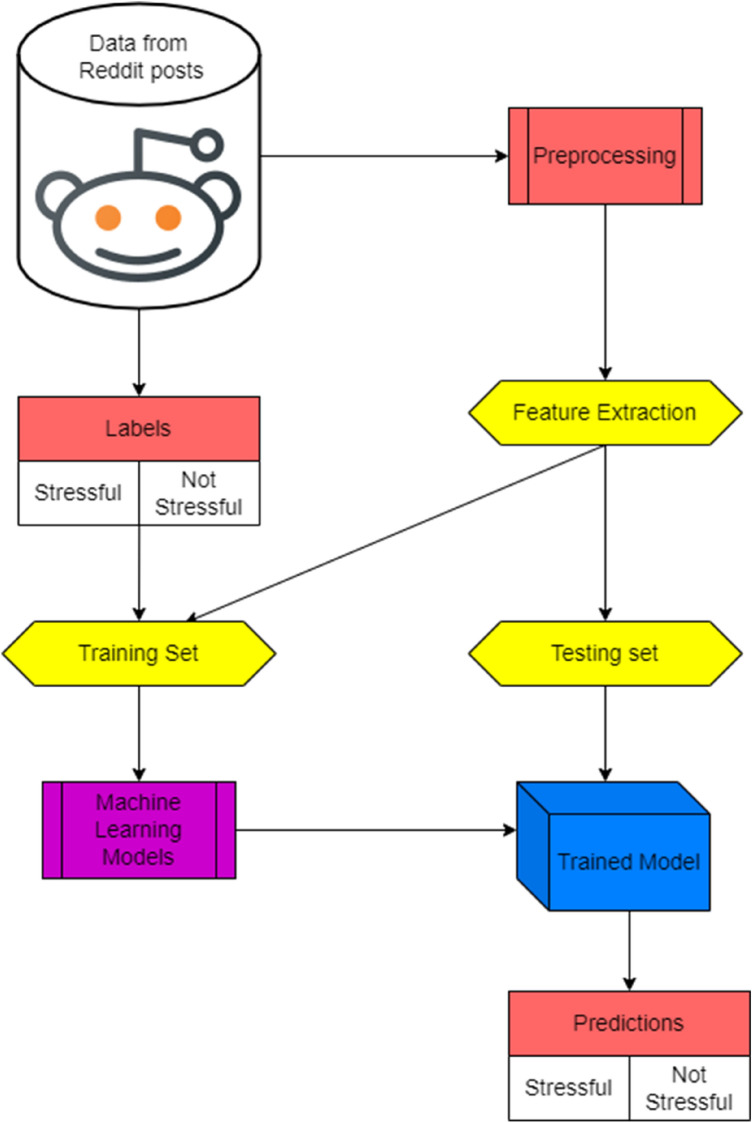
Proposed architecture diagram

Figure 1 and Figure 2 depict the commonly occurring words in Stressful and Non-Stressful posts. The posts are labelled as 1 for Stressful and 0 for Non-Stressful. We have used this data with careful consideration of the ethical challenges and recommendations [43]. Fig. 3 Since Reddit is regarded as an anonymous website, the original posters have not been identified during reporting these results.

### Methodology

1. Data-preprocessing- The text content contains much noise, hampering text classification. So, stop words, punctuations, links, direct calls to subreddits (for example, r/psychology), and the likes were removed.
2. Keyword Extraction—After performing keyword extraction, which returns a list of keywords without stop words, all the keywords were combined to make it easier to perform BERT tokenization and ELMo vectorization.
3. Creating Embeddings—The authors used the ELMo model to create word embeddings and BERT for text tokenization. The authors also considered the cleaned sentences for the BERT tokenization, which consist only of keywords extracted in previous steps.
4. An ELMo model was trained on the cleaned text, forming vectors as word embeddings.
5. Machine learning models—These vectors are then used as inputs for different machine learning algorithms that are trained and whose results were then compared, showing the F1 Scores, Precision, and Recall scores.

To this end, three models such as LR, SVM, and XGBoost were used.

### Data Preprocessing

**Fig. 4:**
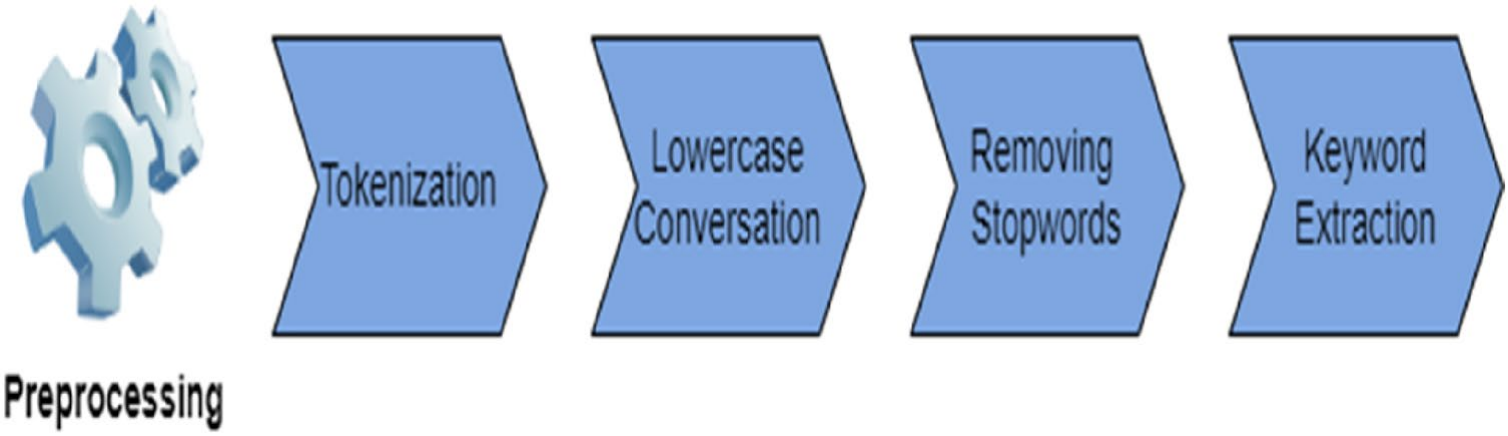
Preprocessing techniques were used

RAKE (Rapid Automatic Keyword Extraction) is a common algorithm used across most applications in natural language processing; this algorithm uses a list of stop words and delimiters to extract relevant phrases and words from a target text [44]. It extracts keywords based on a scoring system which it implements using stop-lists.[45] This algorithm was used in the data preprocessing steps to use only relevant critical phrases as inputs to apply both BERT tokenization and generating ELMo vectors. The data preprocessing steps are shown in Figure 4.

### Embedding Models Used

Here, we used BERT, ELMo, and Bag of Words techniques to create word embeddings from the cleaned text. These embeddings are used to train the ML models. The BERT helps preserve the context of a sequence of text by representing the sentence in the form of vectors. Similarly, ELMo uses character-level embeddings to create vectors for sequences. Bag-of-Words, however, disregard grammar and order of words and represent the sentence in the manner of a word count.

## Results and Discussions

### Performance Metrics

The metrics used to measure the performance of our models are Precision, Recall, and F-scores [11] and are represented in equations (1–3). The results of these metrics are represented in Table 1.

**Table 1:** Examples of Stressful and Non-Stressful posts

Subreddit	Text	Label	Post ID
r/ptsd	He said he had not felt that way before and suggested I go rest, so..TRIGGER AHEAD IF YOU’RE A HYPOCONDRIAC LIKE ME: i decide to look up “feelings of doom” …	1	8601tu
r/relationships	until i met my new boyfriend, he is amazing, he is kind, he is sweet, he is a good student, he likes the same things as me, my family likes him, and so on, but i don’t feel that passion that rush i felt …	1	7rorpp
r/domesticviolence	It was a big company, so luckily I didn't have to see him all the time, but when I did, he again acted as though I didn’t exist. I tried to talk to him and update him on the pregnancy…	0	7iphly

1 $$p = \frac{TP}{(TP + FP)}$$

2 $$r = \frac{TP}{(TP + FN)}$$

3 $$F1 = \frac{TP}{(TP+(\frac{FP + FN}{2}))}$$

We used the Rapid Automatic Keyword Extraction algorithm (RAKE) to extract keywords from the texts in our dataset [37]. These keywords were then encoded using ELMo vectors which form contextualized encodings of text strings in each post. Compared to the BERT encoder, which tokenizes and encodes the texts in the dataset, Elmo has more features as every keyword has 1024 parameters. The feature list formed using the Elmo encoder creates an array of shapes [n, m, 1024], where n is the number of sentences and m is the maximum number of words in each sentence.

Next, we used supervised machine learning models with different feature extraction methods. The paper experiments with ELMo vectors, BERT vectors, Bag-of-Words, and weighted TFIDF vectors to form features to train ML models for optimum performance. The results are presented in table 2.

**Table 2:** Precision, Recall and F1 scores for our various experimental models

Embedding Method	Classifier	F1 score	Recall	Average precision	Accuracy
BERT	Logistic Regression	0.56	0.53	0.55	0.53
	XGBoost	0.68	0.53	0.56	0.56
	SVM	0.64	0.56	0.57	0.57
BoW*TF-IDF	Logistic Regression	0.70	0.60	0.64	0.65
	XGBoost	0.60	0.66	0.63	0.66
	SVM	0.69	0.64	0.60	0.64
ELMO	Logistic Regression	0.76	0.74	0.70	0.74
	XGBoost	0.70	0.61	0.62	0.63
	SVM	0.76	0.74	0.70	0.74

It is observed that LR, SVM, and XGBoost give better results than other test classifiers. An F1 score of 0.76 was achieved using LR and ELMo embeddings trained on the labeled dataset. Compared to SVM and the XGBoost model, Logistic Regression has the highest F1 score. With BERT embeddings, the same algorithms yielded F1 scores of 0.56, 0.64, and 0.68, respectively. The XGBoost model displayed higher scores for different contextual embeddings.

**Fig. 5:**
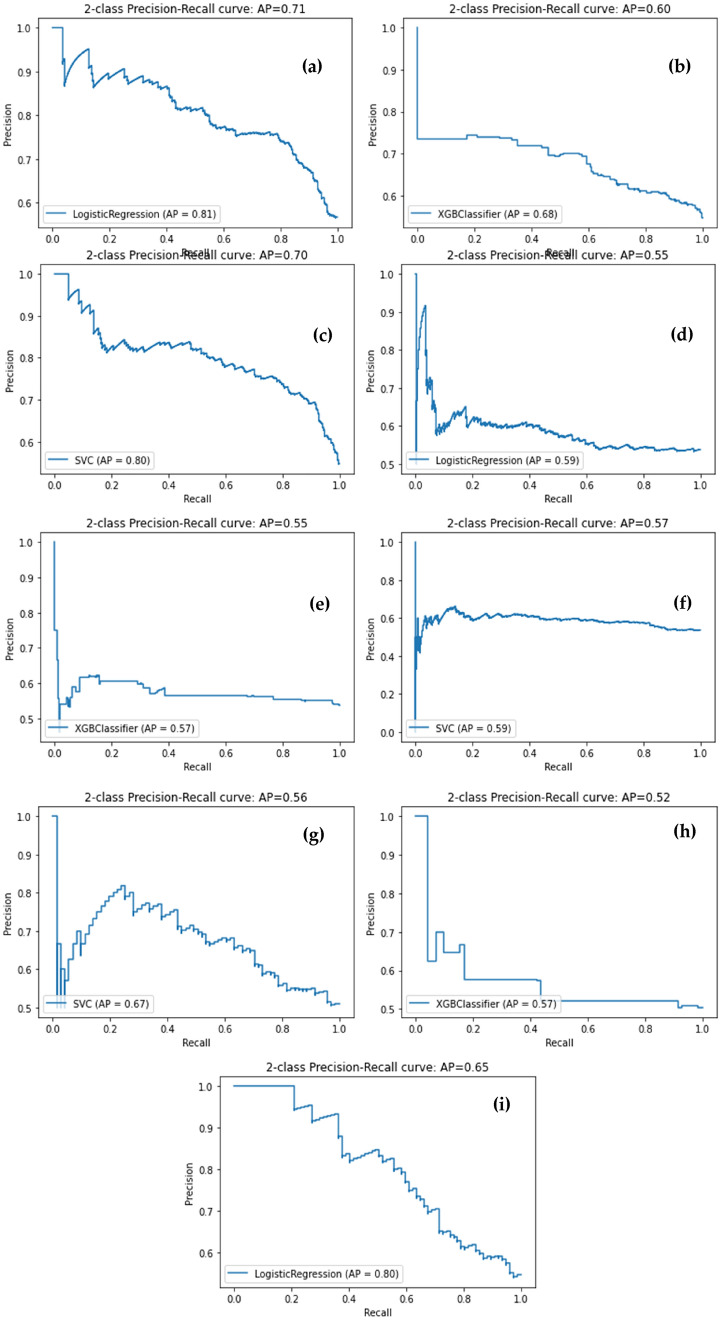
Precision—Recall curve for results of: **a** Logistic Regression classifier with ELMO embeddings; **b** XGBoost classifier with ELMO embeddings; **c** SVM classifier with ELMO embeddings; **d** Logistic Regression classifier with BERT embeddings; e XGBoost classifier with BERT embeddings; f SVM classifier with BERT embeddings; g SVM classifier with BoW vectors + TF-IDF; **h** XGBoost classifier with BoW vectors + TF-IDF; and **i** Logistic Regression with BoW vectors + TF-IDF

This paper also considers experiments with bag-of-words embeddings with weighted TF-IDF vectors to form features for the ML models. This embedding method yielded the highest F1 score of 0.70. The precision scores using Logistic Regression, XGBoost model, and SVM were 0.70, 0.60, and 0.69, respectively. The Precision-Recall curves for the three models are represented in Figure 5.

### Precision-Recall Graphs

This section discusses the results of the various supervised machine learning algorithms and models used. Figure 5 contains the precision-recall graphs that visually describe the algorithm's training and performance. It summarizes information about these performance metrics in a single pictorial representation.

The results revealed that the LR model combined with ELMo vectors provided the best results in classifying whether the post shows signs of mental stress. The performance of LR and SVM is comparable in the case of all three types of embeddings. The two algorithms differ in their loss functions. LR minimizes logistic loss, whereas the SVM employs a hinge loss function [27].

The difference in accuracy, precision, and recall depends on the underlying mathematics and the stochastic nature of each algorithm. Due to the algorithms being stochastic, minor differences can be seen each time the model is trained with the same algorithm [40]. Different values were set for each hyperparameter when the dataset was shuffled. The recorded values, which are used for calculating and minimizing loss, change depending on the order in which the data points are analyzed. This is one of the few reasons a dataset is shuffled.

In LR model, the hyperparameters are the significant contributors to variations in performance metrics during backpropagation. A learning rate is applied in the gradient descent function, uses the cost function, and tries to achieve a minimum on the loss curve [46]. This also means that the metrics heavily depend on the generated cost function. The variations can then be explained by the initial conditions of the training process. The highest F1 score achieved for the LR model is 0.76, as seen in Table 2. XGBoost is an ensemble algorithm that uses the concept of decision trees in the initial stages of the training process. Along with that, some advanced mathematical techniques are developed that aid the metrics of XGBoost. The gradient boosting method applied here creates new models to evaluate the errors from the previous models created by this algorithm. This contributes significantly to the high-performance metrics XGBoost generally showcase in various applications. The highest F1 score achieved for XGBoost is 0.70. Table 3 presents the supervised learning methods used by Turcan et al. [2], when evaluating baseline scores trained over various subsets of the dataset.

**Table 3:** Comparison with other benchmark papers for the same dataset [2]

Model	P	R	F
Majority baseline	0.5161	1.0000	0.6808
CNN + features	0.6023	0.8455	0.7035
CNN	0.5840	0.9322	0.7182
GRNN w/ attention + features	0.6792	0.7859	0.7286
GRNN w/ attention	0.7020	0.7724	0.7355
N-gram baseline	0.7249	0.7642	0.7441
N-gram + features	0.7474	0.7940	0.7700
LogReg w/ pre trained Word2Vec + features	0.7346	0.8103	0.7706
LogReg w/ fine-tuned BERT LM + features	0.7704	0.8184	0.7937
LogReg w/ domain Word2Vec + features	0.7433	0.8320	0.7980
BERT-base	0.7518	0.8699	0.8065

The LSTM model used by ELMO and the positional embeddings created by the BERT model help increase the accuracy of these models by extracting essential and highly relevant unique features from the text. In the SVM model, the performance metrics are governed by how distinguishable the different dataset classes are. SVMs use the concept of hyperplanes to separate the different classes, and as a result, the metrics are affected by how well they are separable [47]. The support vector classifier achieved an F1 score of 0.76 in this paper’s experiments. The previous baseline results obtained by Turcan et al., 2019 are shown in Table 2. Compared to their baseline scores, the models used in pair with the various embeddings methods have proven to provide high results and increased accuracy.

As shown in Table 2, the results show that LR and SVM showed the highest results in terms of this dataset. It is also observed that the usage of ELMo vectors and BERT embeddings results in higher scores than the Bag-of-Words model; this can be attributed to the preservation of the context of words by the ELMo model, which leads to higher accuracy for classification.

The findings generated by this study can establish a foundation for our subsequent research which encompass leveraging pre-trained models [48–51] to improve performance and establish benchmarks for long-form textual content in line with those established for shorter form textual content [52].

## Limitations

The small dataset size proved to be a fundamental limitation in achieving higher accuracy for our models. The data used has captured users’ posts on a single platform in recognizing stress. While this proves to be accurate for the given post, analyzing posts made by authors on multiple platforms can give us a better sense of the mental state of the authors of the post.

Despite being limited due to the smaller size of the dataset, this paper achieves its goal of successfully identifying stressful posts and providing significant results using standard evaluation metrics. The data from the source was collected using the PRAW API, which could extract more data from targeted subreddits to perform deeper stress analysis with the help of the technologies described in this paper.

Sentiment analysis has always been an exciting research topic amongst NLP researchers. Similar research can be applied to different applications like Human-Computer Interaction, where it can make the process of communication and interaction much smoother and more straightforward. This will also help improve machine translation. We also see some research in text classification, where texts from various languages have been processed, and the classification has identified the authors of the texts. This has various applications in language identification, detection of spam, and author identification. Opinion mining with NLP and Deep Learning can help academic research and commercial purposes like advertising and product reviewing. Some crucial algorithms used for these research topics are Word2vec, GloVe encodings , and tf-idf vectors for preprocessing of text data.

Different datasets with emotions can be explored for sentiment analysis, and the combination of both text and images that form multimodal sentiment analysis can also be explored. These datasets include blogs, forums, review sites, and the likes. These sources represent the opinions and sentiments of the public and would yield optimum results.

## Conclusion

Mental stress analysis and detection on the internet has been a crucial topic of research in the NLP domain. This spans across many online social media websites, anonymous or otherwise. This paper proposes a method to recognize signs of mental stress in social media posts using machine learning algorithms and natural language processing techniques. By comparing the classification performance of select ML models, the authors have identified the best-performing model for the Dreaddit dataset.

From the results presented, it is evident that the SVM model used along with the ELMo embedding method outperformed other models in terms of F1 score, recall, and average precision. This finding is significant as it highlights the effectiveness of using advanced techniques like ELMo embeddings in combination with traditional models like SVM to improve mental stress analysis in social media. The authors hope that this study provides a foundation for future research exploring neural network-based models and pre-trained language models for mental stress analysis. Additionally, the establishment of benchmarks for Reddit datasets, similar to ones existing for other social media, could prove helpful in categorizing posts into strengths of stress, thus providing further insight into the impact of mental stress on social media users.

Overall, the proposed model has the potential to significantly reduce mental health problems amongst most social media users by identifying signs and providing support and assistance to overcome the same.

## Data Availability

All data generated or analyzed during this study are included in this published article (and its supplementary information files).

## References

[1] American Psychological Association. (2021, March 11). One year of Unhealthy weight gains and increased drinking were reported by Americans coping with pandemic stress [Press release]. http://www.apa.org/news/press/releases/2021/03/one-year-pandemic-stress

[2] Turcan, E., & McKeown, K. (2019, October 31). Dreaddit: A Reddit dataset for stress analysis in Social Media. arXiv.org. Retrieved November 7, 2021, from https://arxiv.org/abs/1911.00133.

[3] Stirman S, Pennebaker J (2001). Word use in the poetry of suicidal and nonsuicidal poets. Psychosom Med.

[4] Zinken J, Zinken K, Wilson J, Butler L, Skinner T (2010). Analysis of syntax and word use to predict successful participation in guided self-help for anxiety and depression. Psychiatry Res.

[5] Rude S, Gortner E-M, Pennebaker J (2004). Language use of depressed and depression-vulnerable college students. Cogn Emot.

[6] Kim J, Lee J, Park E (2020). A deep learning model for detecting mental illness from user content on social media. Sci Rep.

[7] Pirina, I.L., Çöltekin, Ç. (2018). Identifying Depression on Reddit: The Effect of Training Data. EMNLP

[8] Sekulić, Ivan , Strube, Michael. (2020). Adapting Deep Learning Methods for Mental Health Prediction on Social Media.

[9] Fabris MA, Marengo D, Longobardi C, Settanni M (2020). Investigating the links between fear of missing out, social media addiction, and emotional symptoms in adolescence: the role of stress associated with neglect and negative reactions on social media. Addictive Behaviors.

[10] Brailovskaia J, Schillack H, Margraf J (2020). Tell me why you are using social media (SM)! Relationship between reasons for the use of SM, SM flow, daily stress, depression, anxiety, and addictive SM use – an exploratory investigation of young adults in Germany. Computers in Human Behavior.

[11] Guntuku SC, Yaden D, Kern M, Ungar L, Eichstaedt J (2017). Detecting depression and mental illness on social media: an integrative review. Curr Opin Behav Sci.

[12] Lin, H., Jia, J., Guo, Q., Xue, Y., Li, Q., Huang, J., Cai, L., Feng, L., (2014). User-level psychological stress detection from social media using deep neural network. MM 2014 - Proceedings of the 2014 ACM Conference on Multimedia 10.1145/2647868.2654945

[13] Peters, M.E., Neumann, M., Iyyer, M., Gardner, M., Clark, C., Lee, K., Zettlemoyer, L. (2018). Deep Contextualized Word Representations. NAACL.

[14] Devlin, J., Chang, M., Lee, K., & Toutanova, K. (2019). BERT: Pre-training of Deep Bidirectional Transformers for Language Understanding. NAACL.

[15] Rogers A, Kovaleva O, Rumshisky A (2021). A Primer in BERTology: what we know about how BERT works. Trans Association Computational Linguistics.

[16] D’Sa, A. G., Illina, I., Fohr, D. (2020). BERT and fastText Embeddings for Automatic Detection of Toxic Speech. International Multi-Conference on: “Organization of Knowledge and Advanced Technologies” (OCTA) 10.1109/OCTA49274.2020.9151853

[17] Teng Li; Tao Mei; In-So Kweon; Xian-Sheng Hua (2011). Contextual Bag-of-Words for Visual Categorization. 21 (4) 381–392.

[18] Yin Zhang; Rong Jin; Zhi-Hua Zhou (2010). Understanding bag-of-words model: a statistical framework. 1 (1–4), 43–52. 10.1007/s13042-010-0001-0

[19] Akiko Aizawa (2003). An information-theoretic perspective of tf–idf measures. 39 (1), 45–65. 10.1016/s0306-4573(02)00021-3

[20] Wu, Ho Chung; Luk, Robert Wing Pong; Wong, Kam Fai; Kwok, Kui Lam (2008). Interpreting TF-IDF term weights as making relevance decisions. 26 (3), 1–37. 10.1145/1361684.1361686

[21] Pedregosa, Fabian & Varoquaux, Gael & Gramfort, Alexandre & Michel, Vincent & Thirion, Bertrand & Grisel, Olivier & Blondel, Mathieu & Prettenhofer, Peter & Weiss, Ron & Dubourg, Vincent & Vanderplas, Jake & Passos, Alexandre & Cournapeau, David & Brucher, Matthieu & Perrot, Matthieu & Duchesnay, Edouard & Louppe, Gilles. (2012). Scikit-learn: Machine Learning in Python. Journal of Machine Learning Research. 12.

[22] Anthony J. Myles; Robert N. Feudale; Yang Liu; Nathaniel A. Woody; Steven D. Brown (2004). An introduction to decision tree modeling.18 (6), 275–285. 10.1002/cem.873

[23] Yong Z, Youwen L, Shixiong X (2009). An improved KNN Text classification algorithm based on clustering. Journal Computers.

[24] Meersman, Robert; Tari, Zahir; Schmidt, Douglas C. (2003). [Lecture Notes in Computer Science] On the Move to Meaningful Internet Systems 2003: CoopIS, DOA, and ODBASE Volume 2888 || KNN Model-Based Approach in Classification. 10.1007/978-3-540-39964-3_62

[25] Gayathri, K.; Marimuthu, A. (2013). [IEEE 2013 7th International Conference on Intelligent Systems and Control (ISCO) - Coimbatore, Tamil Nadu, India (2013.01.4–2013.01.5)] 2013 7th International Conference on Intelligent Systems and Control (ISCO) - Text document preprocessing with the KNN for classification using the SVM. 453–457. 10.1109/ISCO.2013.6481197

[26] Noble, William S (2006). What is a support vector machine?. 24 (12), 1565–1567. 10.1038/nbt1206-1565 10.1038/nbt1206-1565 17160063

[27] Lilleberg, Y. Zhu and Y. Zhang, (2015) “Support vector machines and Word2vec for text classification with semantic features,” IEEE 14th International Conference on Cognitive Informatics Cognitive Computing (ICCI*CC), 10.1109/ICCI-CC.2015.7259377.

[28] Ren X., Guo H., Li S., Wang S., Li J. (2017) A Novel Image Classification Method with CNN-XGBoost Model. In: Kraetzer C., Shi YQ., Dittmann J., Kim H. (eds) Digital Forensics and Watermarking. IWDW 2017. Lecture Notes in Computer Science, vol 10431. Springer, Cham. 10.1007/978-3-319-64185-0_28

[29] Ogunleye and Q. -G. Wang, (2020) “XGBoost Model for Chronic Kidney Disease Diagnosis,” in IEEE/ACM transactions on computational biology and bioinformatics.10.1109/TCBB.2019.2911071 10.1109/TCBB.2019.2911071 30998478

[30] Kartik Budholiya, Shailendra Kumar Shrivastava, Vivek Sharma, (2020) An optimized XGBoost based diagnostic system for effective prediction of heart disease,Journal of King Saud University - Computer and Information Sciences

[31] Chen, T., Guestrin, C. (2016). XGBoost: A Scalable Tree Boosting System. Proceedings of the 22nd ACM SIGKDD International Conference on Knowledge Discovery and Data Mining. Presented at the San Francisco, California, USA

[32] Gortmaker L (1994). ‘Theory and methods–applied logistic regression by david w hosmer jr and stanley lemeshow’. Contemp Sociol.

[33] Indra T, Wikarsa L, Turang R (2016). Using logistic regression method to classify tweets into the selected topics. Intern Confer Adv Com Sci Inform Syst (ICACSIS).

[34] Jia, J., (2018) January. Mental Health Computing via Harvesting Social Media Data. In IJCAI 5677–5681.

[35] Turcan, E., Muresan, S., & McKeown, K. (2021, Junie). Emotion-Infused Models for Explainable Psychological Stress Detection. Proceedings of the 2021 conference of the North American chapter of the association for computational linguistics: human language technologies 10.18653/v1/2021.naacl-main.230

[36] Pay, T., Lucci, S., Cox, J.L. (2019). An Ensemble of Automatic Keyword Extractors: TextRank, RAKE and TAKE. Computación y Sistemas 23.

[37] Rose, S., Engel, D., Cramer, N., Cowley, W. (03 2010). Automatic Keyword Extraction from Individual Documents. 10.1002/9780470689646.ch1

[38] Goutte C, Gaussier E (2005). A Probabilistic interpretation of precision, recall and F-score, with implication for evaluation. Lect Notes Comput Sci.

[39] Yichao Wu, Liu Yufeng (2007). Robust truncated hinge loss support vector machines. J Am Statistical Association.

[40] Walker S, Khan W, Katic K, Maassen W, Zeiler W (2020). Accuracy of different machine learning algorithms and added-value of predicting aggregated-level energy performance of commercial buildings. Energy Buildings.

[41] Ewout W Steyerberg; Frank E Harrell Jr; Gerard J.J.M Borsboom; M.J.C Eijkemans; Yvonne Vergouwe; J.Dik F Habbema (2001). Internal validation of predictive models: Efficiency of some procedures for logistic regression analysis. 10.1016/s0895-4356(01)00341-9 10.1016/s0895-4356(01)00341-9 11470385

[42] Alhanoof A, Duaa A, Heyam A, Amani S, Alanoud D, Najla A, Elwafa A, Heba AK (2021). Impact of dataset size on classification performance: an empirical evaluation in the medical domain. Applied Sciences..

[43] Brown, R. D. (1990). Human-computer interaction for semantic disambiguation. In COLING 1990 Volume 3: Papers presented to the 13th International Conference on Computational Linguistics.

[44] Parcheta Z, Sanchis-Trilles G, Casacuberta F (2021). Combining embeddings of input data for text classification. Neural Process Lett.

[45] Liu B (2012). Sentiment analysis and opinion mining. Synthesis Lect Human Lang Technolo.

[46] Pennington, J., Socher, R., & Manning, C. (2014, Oktober). GloVe: Global Vectors for Word Representation. Proceedings of the 2014 Conference on Empirical Methods in Natural Language Processing (EMNLP) 10.3115/v1/D14-1162

[47] Turcan, E., Muresan, S., & McKeown, K. (01 2021). Emotion-Infused models for explainable psychological stress detection.10.18653/v1/2021.naacl-main.230

[48] Sanh, V., Debut, L., Chaumond, J., & Wolf, T. (2019). DistilBERT, a distilled version of BERT: smaller, faster, cheaper and lighter. CoRR, abs/1910.01108. Ανακτήθηκε από http://arxiv.org/abs/1910.01108

[49] Ji, S., Zhang, T., Ansari, L., Fu, J., Tiwari, P., & Cambria, E. (2021). MentalBERT: Publicly Available Pretrained Language Models for Mental Healthcare. 10.48550/ARXIV.2110.15621

[50] Naseem, U., Lee, B. C., Khushi, M., Kim, J., & Dunn, A. G. (2022). Benchmarking for Public Health Surveillance tasks on Social Media with a Domain-Specific Pretrained Language Model. 10.48550/ARXIV.2204.04521

[51] Winata, G. I., Kampman, O. P., & Fung, P. (2018, Απρίλιος). Attention-Based LSTM for Psychological Stress Detection from Spoken Language Using Distant Supervision. 2018 IEEE International Conference on Acoustics, Speech, and Signal Processing (ICASSP). 10.1109/icassp.2018.8461990

[52] Thelwall M (2017). TensiStrength: Stress and relaxation magnitude detection for social media texts. Inf Process Manage.

